# Evaluate the immune-related eRNA models and signature score to predict the response to immunotherapy in thyroid carcinoma

**DOI:** 10.1186/s12935-022-02722-8

**Published:** 2022-10-10

**Authors:** Pu Wu, Jinyuan Shi, Zhiyuan Wang, Wei Sun, Hao Zhang

**Affiliations:** Department of Thyroid Surgery, The First Hospital of China Medical University, Shenyang, China

**Keywords:** eRNAs, Tumor immune microenvironment, THCA, Prognostic signature, Immune checkpoints

## Abstract

**Background:**

The functional alterations of eRNAs have been reported to be correlated with tumorigenesis. However, the roles of eRNAs in thyroid cancer (THCA) remain still unclear. This study aimed to construct an immune-related eRNA prognostic signature that could effectively predict the survival and prognosis for THCA.

**Methods:**

The Weighted Gene Co-Expression Network Analysis (WGCNA) was performed to identify THCA-specific immune-related hub genes and immune-related eRNAs were obtained using Pearson correlation analysis. Univariate and least absolute shrinkage and selection operator (LASSO) Cox regression were conducted to construct an immune-related eRNA prognostic signature in training cohort, and the predictive capability was verified in test cohort and entire cohort. Kaplan–Meier analysis, principal component analysis (PCA), receiver operating characteristic (ROC) curves, and nomogram were used to validate the risk signature. Furthermore, CIBERSORT, ESTIMATE and ssGSEA were analyzed to explore the tumor immune microenvironment (TIME) of the risk signature, and the response of potential immunotherapeutic were also discussed.

**Results:**

A total of 125 immune-related eRNAs were obtained and 16 immune-related eRNAs were significantly correlated with overall survival (OS). A 9-immune-related eRNA prognostic signature was constructed, and the risk score was identified as an independent predictor. High-risk groups were associated with a poorer OS. Immune microenvironment analysis indicated that low risk score was correlated with higher immuneScore, high immune cell infiltration, and the better response of immunotherapy. Additionally, we also detected 9 immune-related eRNA expression levels in sixty-two matched tumorous and non-tumorous tissues using qRT-PCR analysis.

**Conclusion:**

Our immune-related eRNA risk signature that was an independent prognostic factor was strongly correlated with the immune microenvironment and may be promising for the clinical prediction of prognosis and immunotherapeutic responses in THCA patients.

**Supplementary Information:**

The online version contains supplementary material available at 10.1186/s12935-022-02722-8.

## Introduction

The incidence of thyroid cancer (THCA) is increasing steadily in the past few decades [1]. THCA is the most common malignancy of the endocrine system, it can be classified into four main histological types: well-differentiated papillary thyroid carcinoma (PTC), follicular thyroid cancer (FTC), medullary thyroid cancer (MTC) and undifferentiated or anaplastic thyroid cancer (ATC) [2]. PTC is the most predominant subtype and accounts for more than 85% among all the THCA cases. Although the majority of PTC patients usually have an excellent overall prognosis after surgery and radioactive iodine therapy, regional neck lymph node metastasis (LNM) in advanced PTC is associated with local recurrence and distant metastasis, and this has been reported as an independent factor of a poor prognosis [3, 4]. Therefore, it is urgent to explore and identify sensitive prognostic biomarkers for THCA to facilitate rational individualized treatment.

The tumor microenvironment (TME) consists of the stromal and immune cells. The constant interactions between tumor cells and the TME play crucial roles in tumor initiation, progression, metastasis, as well as response to therapies [5]. The TME has attracted considerable attention and interest for their potential to consider to be a therapeutic target in various cancers for anti-tumorigenesis treatment [6]. The immune system plays critical roles in tumor formation and progression. A growing body of evidence has indicated that immune-related genes (IRGs) have increasingly been recognized as biomarkers to predict cancer patient prognosis [7]. Presently, some IRG signatures had been constructed to serve as efficiently predictive and prognostic models among hepatocellular carcinoma (HCC) [8, 9], cervical cancer [10], head and neck squamous cell carcinoma (HNSCC) [11, 12], gastric cancer (GC) [13], neuroblastoma (NBL) [14], prostatic adenocarcinoma (PRAD) patients [15].

Long non-coding RNAs (lncRNAs) have gained substantial attention due to their multi-faceted ability to regulate gene expression [16]. Recent studies showed that enhancers were found to be transcriptionally active and generate and transcribe noncoding RNAs, which were known as enhancer RNAs (eRNAs). eRNAs played a fundamental role in tumor initiation, development, and treatment. eRNAs involved in various cancer signaling pathways through regulating their target genes. Oncogene-induce eRNAs can directly contribute to tumorigenesis. In contrast, tumor suppressors can also induce eRNAs to suppress tumors. eRNAs have emerged as important regulators of the immune response, and played a central role in controlling immune-related functions [17]. Furthermore, eRNAs could also regulate clinically actionable genes and immune checkpoints, which indicated the potentially clinical utility of eRNAs in cancer therapy. Unfortunately, there are limited studies focusing on immune-related eRNAs and the potential prognostic value of immune-related eRNAs in THCA remains unclear; therefore, effective prognostic biomarkers for THCA are urgently needed.

In this study, we aimed to identify immune-related eRNAs using Pearson correlation analysis and construct immune-related eRNAs prognostic signature to systematically explore the prognostic value of the risk signature in TCGA patients from TCGA database. We then further investigated the associations between prognostic signature and clinicopathological factors, tumor immune microenvironment (TIME), immunotherapy responses. Furthermore, a nomogram was established to predict the OS of THCA patients. Our study may provide a new insight to predict the potential response for immunotherapy for THCA patients.

## Materials and methods

### Data extraction

The RNA sequencing transcriptome data (58 normal samples and 510 THCA samples) and related clinical information of THCA were downloaded from The Cancer Genome Atlas (TCGA) database and Genotype-Tissue Expression Project (GTEx) database. The clinical characteristics of all THCA patients were listed in Table 1. The immune-related genes (IRGs) were downloaded from the Immunology Database and Analysis Portal (ImmPort, https://www.immport.org/) and InnateDB (https://www.innatedb.com/) databases.

**Table 1:** Patients’ clinical characteristics of training, test and entire cohorts

Variables	Total cohort %	Training cohort %	Test cohort %	*p*-value
Age				
< = 60	389 (77.49)	190 (75.7)	199 (79.28)	0.3926
> 60	113 (22.51)	61 (24.3)	52 (20.72)	
Gender				
Female	367 (73.11)	188 (74.9)	179 (71.31)	0.4207
Male	135 (26.89)	63 (25.1)	72 (28.69)	
Stage				
Stage I–II	333 (66.33)	158 (62.95)	175 (69.72)	0.1003
Stage III–IV	167 (33.27)	93 (37.05)	74 (29.48)	
Unknow	2 (0.4)	0 (0)	2 (0.8)	
T stage				
T1-2	307 (61.16)	154 (61.35)	153 (60.96)	1
T3-4	193 (38.45)	96 (38.25)	97 (38.65)	
Unknow	2 (0.4)	1 (0.4)	1 (0.4)	
M stage				
M0	282 (56.18)	139 (55.38)	143 (56.97)	1
M1	9 (1.79)	4 (1.59)	5 (1.99)	
Unknow	211 (42.03)	108 (43.03)	103 (41.04)	
N stage				
N0	229 (45.62)	111 (44.22)	118 (47.01)	0.7806
N1	223 (44.42)	112 (44.62)	111 (44.22)	
Unknow	50 (9.96)	28 (11.16)	22 (8.76)	

### Differentially expressed immune-related genes (DE-IRGs)

The differentially expressed genes (DEGs) were identified using “limma” package in the R software (version 4.0.4) between tumor samples and normal specimens and visualized by the heatmap and volcano plot according to the screening criterion of |log_2_ fold change (FC)| > 1.0 and false discovery rate (FDR) value < 0.05 in the TCGA dataset18. Differentially expressed immune-related genes (DE-IRGs) were extracted from DEGs. The visual Venn diagram was constructed by the online tool to show the intersection of the DEGs and IRGs.

### Weighted gene co-expression network analysis (WGCNA)

WGCNA was performed to identify THCA-specific immune genes related to the co-expression modules using “WGCNA” R package based on the DE-IRGs [19]. The Aij = |Sij|^β^ (Aij: adjacency matrix between gene i and gene j, Sij: similarity matrix made by Pearson’s correlation coefficient of all pairs of genes, and β: soft thresholding value) was used to show the weighted adjacency matrix with a scale-free co-expression network and then transformed into a topological overlap matrix (TOM), and gene modules were identified [20]. We calculated the module eigengene (ME) of each module to identify the most significant module. Finally, the module that was highly correlated with THCA was selected for subsequently analysis.

### Identification of immune-related eRNAs in THCA

The eRNAs were predicted according to the PreSTIGE algorithm as previously descirbed [21, 22]. The expression of 1565 eRNAs among 1584 eRNAs were retrieved from the TCGA-THCA samples. The relationship between the eRNAs and IRGs was explored to identify immune-related eRNAs using correlation analysis (|R| > 0.4, P < 0.001).

### Construction and validation of the immune-related eRNAs prognostic signature

All patients were randomly divided into a training cohort (n=251) and a validation cohort (n=251) at 1:1 ratio. The training cohort was used for constructing the immune-related eRNAs prognostic signature, and the predictive performance was verified in the test cohort and total cohort. The Univariate Cox proportional hazard regression analysis was conducted to identify the immune-related eRNAs with prognostic value in the training cohort, and the filter p-value was set at 0.05. The prognostic model was subsequently established using the Least Absolute Shrinkage and Selection Operator (LASSO) penalized Cox proportional hazards regression with “glmnet” R package [23]. The model was determined by penalty parameter (λ) with ten-fold cross-validation following the minimum criteria. The risk score of each THCA patient was calculated by the following formula: Risk score = βA* Expression level of Gene A + βB* Expression level of Gene B + … + βN* Expression level of Gene N, (β: regression coefficient) [24]. Patients were separated into high and low-risk group based on the median risk score. The principal component analysis (PCA) was performed for two risk groups using “scatterplot3d” R package [25]. Kaplan–Meier survival curves were constructed between two risk groups using “survival” R package and the time-dependent receiver operational feature curves (ROC) were carried out with the “survival ROC” R package to verify the sensitivity and specificity of the signature in all cohorts [26]. Univariate and multivariate cox regression analyses were used to determine the independent prognostic factor for OS in all cohorts.

### Development of prognostic nomogram

The nomogram was constructed based on the risk score and clinical features (age, gender, and pathological stage) to predict the survival risk of THCA patients using “rms” R package. The calibration curves were used for comparing the consistency between the predicted and actual survival to evaluate the predictive probability of the nomogram.

### Functional enrichment analysis

The DEGs between the high- and low-risk groups were identified according to the filtering criteria (|log2FC| ≥ 1 and FDR < 0.05). The Gene Ontology (GO) analysis was conducted to explore the potential biological processes based on these DEGs using the “clusterProfifiler” R package. Gene Set Enrichment Analysis (GSEA) was performed to analyze the difference of the immune response between different risk groups using GSEA 4.1.0. A NOM p-value < 0.05 was defined as statistically significant.

### Assessment of immune cell infiltration in THCA

The infiltration levels of immune cells in THCA were estimated based on the gene expression profiles using CIBERSORT algorithm [27]. The differential infiltrating levels of immune cells in high- and low-risk groups were evaluated by the Wilcoxon rank-sum test. Furthermore, the tumor microenvironment score (tumor purity, immune score, stromal score and estimate score) was calculated using the “ESTIMATE” R package [28].

To determine the association between the risk score and immune status, single-sample gene set enrichment analysis (ssGSEA) was performed to calculate the infiltration abundance of 16 immune cells and the activity of 13 immune-related pathways by utilizing the “GSVA” R package [29].

### Immunophenoscore analysis

Immunophenoscore (IPS) was estimated based on the expression of the four determining components of immunogenicity, including effector cells, immunosuppressive cells, major histocompatibility complex (MHC) molecules, and immunomodulators, which can well predict the response to immune checkpoint inhibitors (ICIs). IPS was calculated with a scale ranging from 0 to 10 according to representative cell-type gene expression Z-scores. The IPS of THCA patients were downloaded from The Cancer Immunome Atlas (TCIA) (https://tcia.at/home) [30].

### Quantitative real-time PCR

Sixty-two pairs of PTC tumorous and adjacent normal tissue specimens were collected from the First Affiliated Hospital of China Medical University. The clinicopathological characteristics of 62 THCA patients from our hospital were displayed in Table 2. Total RNA was extracted from tissue samples using RNAiso (Takara, Dalian, China), then RNA was reverse transcribed into cDNA with the QuantiTect Reverse Transcription Kit (Takara, Shiga, Japan). Quantitative Real-Time PCR (qRT-PCR) analyses were performed with SYBR-Green (Takara, Shiga, Japan) to validate mRNA expression level, and the level of GAPDH served as an internal control. The relative expression level was calculated based on the comparative Ct (2^−ΔΔCt^) method. The primers’ sequences are listed in Additional file 3: Table S1.

**Table 2:** The clinicopathological factors in 62 PTC patients

Characteristics	Samples(N = 62)	Percentage (%)
Age		
≤ 60	54	87.1
> 60	8	12.9
Gender		
Female	45	72.6
Male	17	27.4
Tumor size		
< 2 cm	43	69.4
≥ 2 cm	19	30.6
Extrathyroidal invasion		
Yes	7	11.3
No	55	88.7
Multicentricity		
Yes	23	37.1
No	39	62.9
Stage		
Stage I–II	56	90.3
Stage III–IV	6	9.7
T		
T1-2	41	66.1
T3-4	21	33.9
N		
N0	18	29.0
N1	44	71.0

## Results

### Screening of THCA-specific DE-IRGs between normal and tumor tissues

**Fig. 1:**
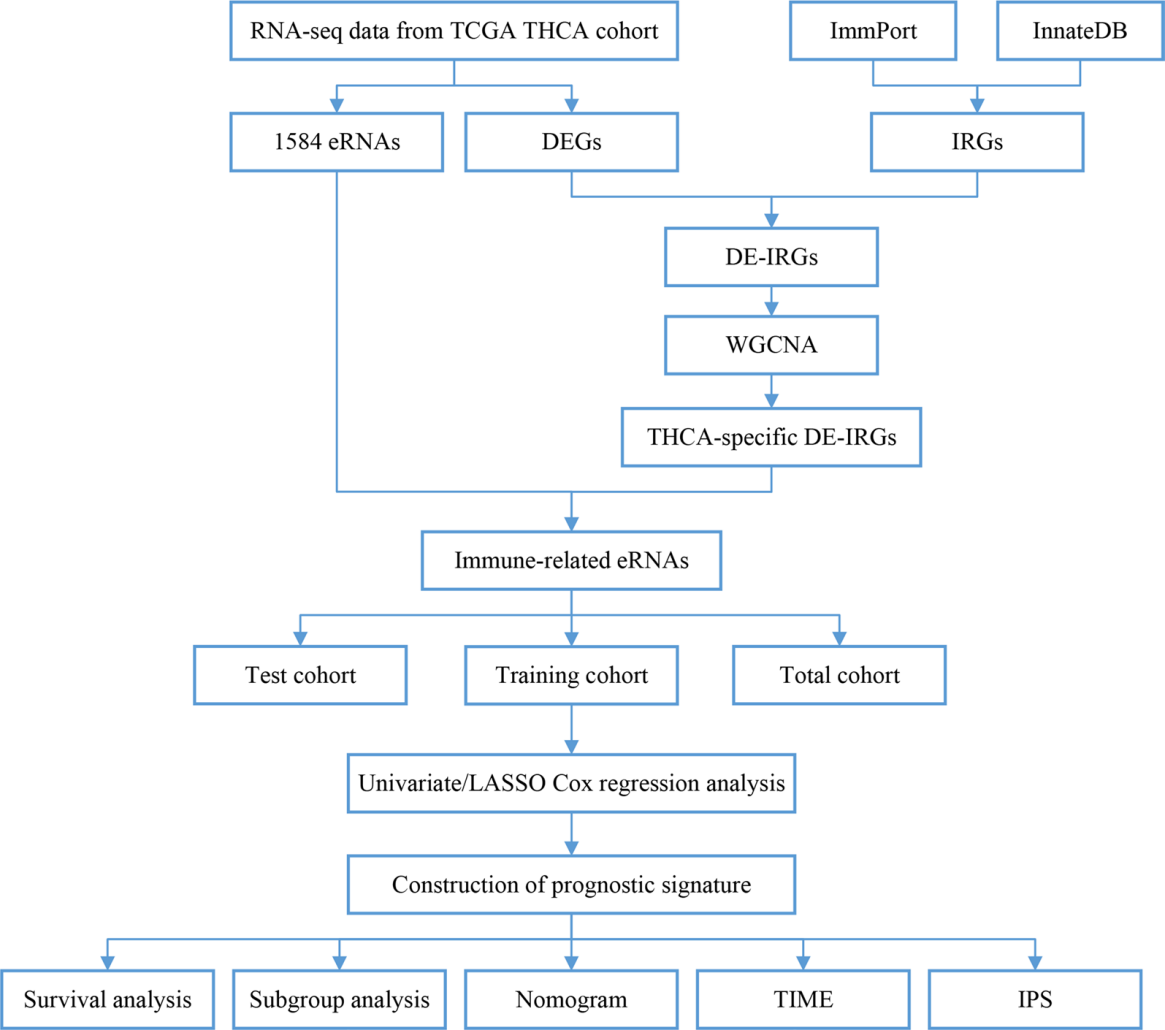
The workflow chart of this study

**Fig. 2:**
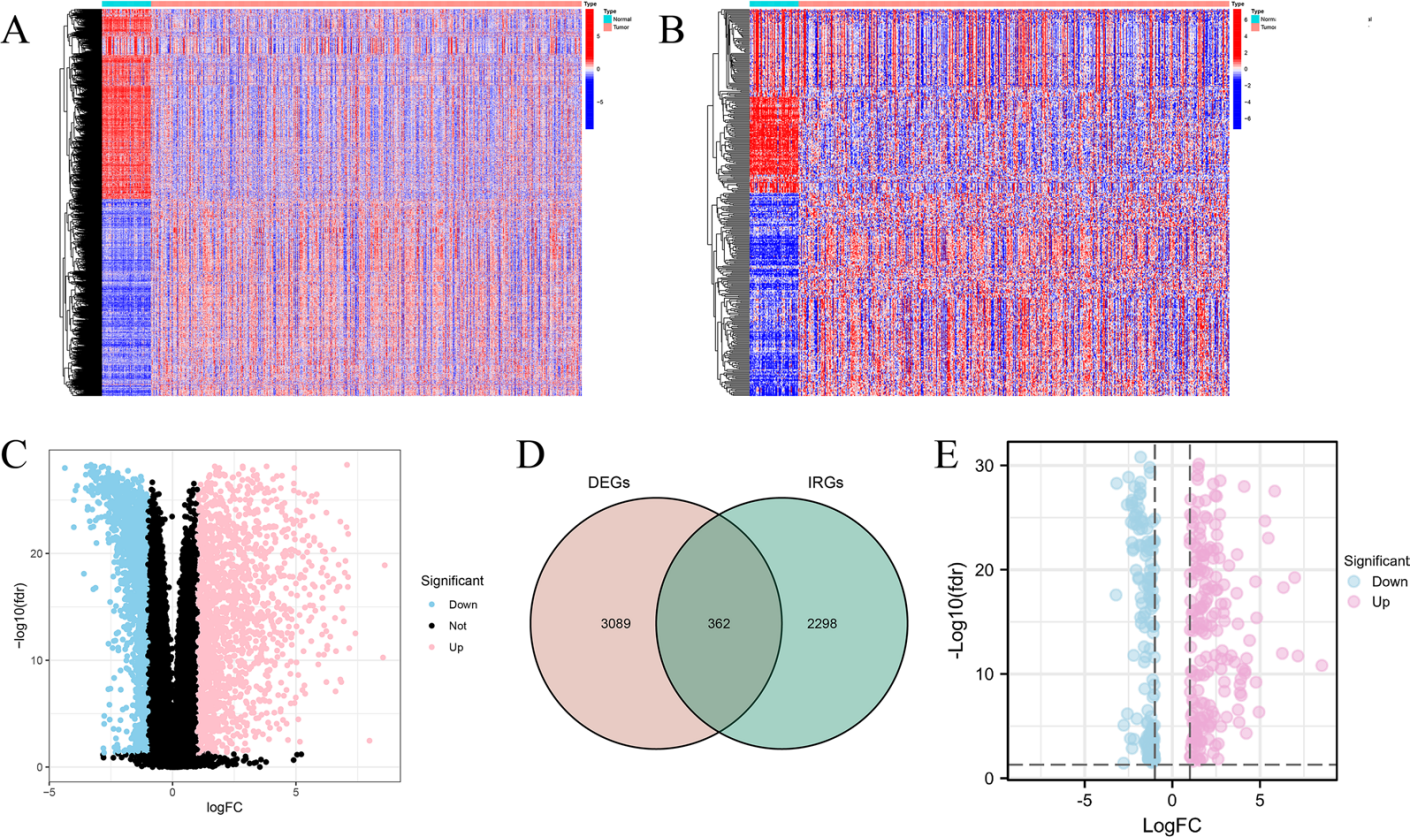
Screening of the differentially expressed immune-related genes in THCA patients. **A** The heatmap of the DEGs between normal and tumor tissues from TCGA database. **B** Heatmap of the DE-IRGs between normal and tumor samples. **C** Volcano plot of DEGs from TCGA database. **D** Venn diagram of the interactions between DEGs and IRGs. **E** Volcano plot of DE-IRGs between normal and tumor tissues

**Fig. 3:**
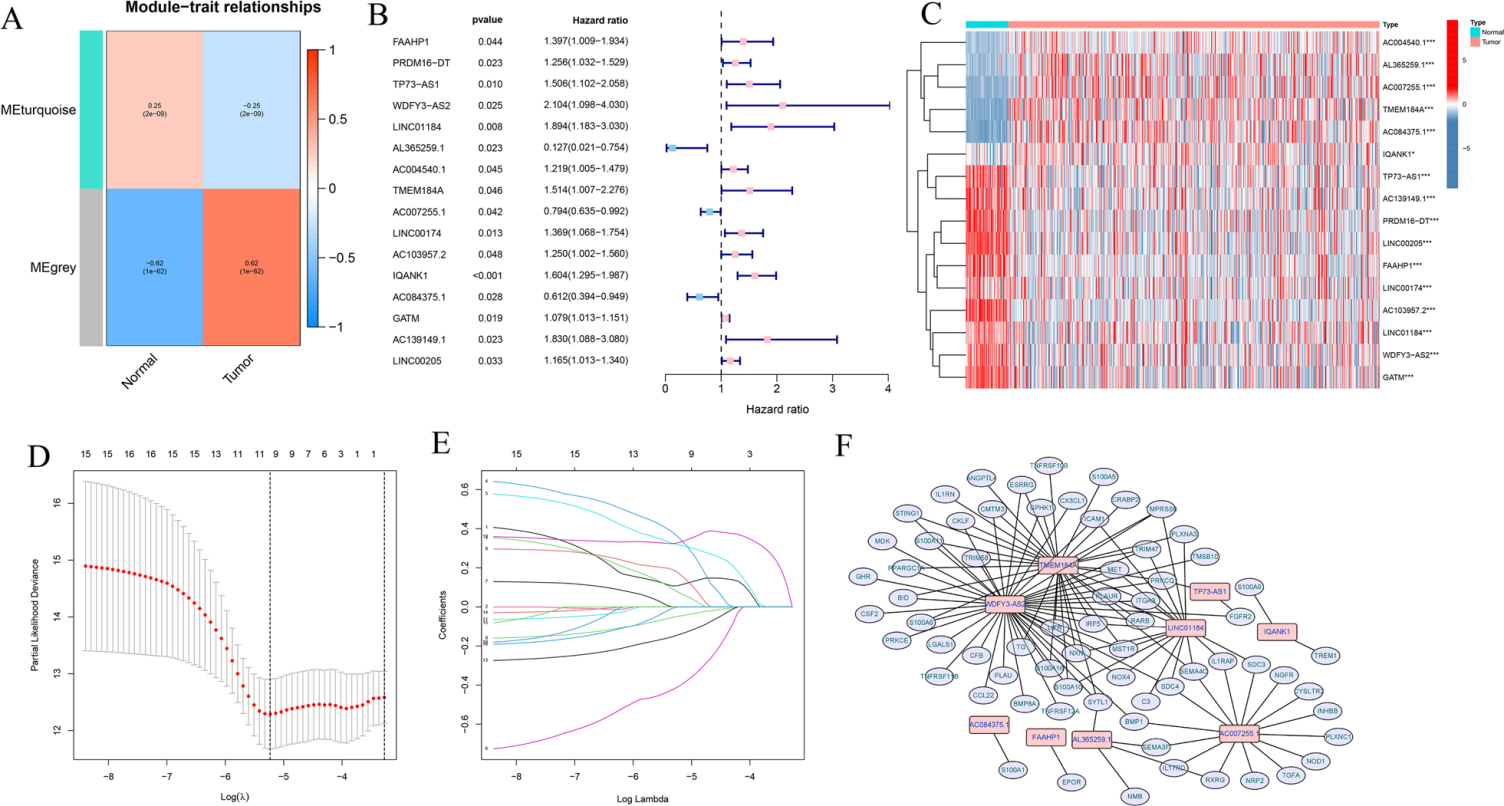
Construction of the immune-related eRNAs prognostic signature for THCA patients. **A** Heatmap of the correlation of gene modules with normal and THCA samples. **B** Univariate Cox regression analysis of immune-related eRNAs in OS. **C** Heatmap of the OS-related genes between normal and tumor tissues. **D**, **E** Construction LASSO regression model based on OS-related immune-related eRNAs in the training cohort. **F** The immune-related eRNAs co-expression network (red: eRNAs, purple: immune genes)

The framework of the analytical process in our study was shown in Fig 1. A total of 3451 DEGs were identified based on the screening criteria of |log_2_(Fold Change) | > 1 and FDR < 0.05, including of 1699 downregulated genes and 1752 upregulated genes (Fig. 2A, C). Additionally, 362 DE-IRGs were extracted from these DEGs (Fig. 2D). Among them, 172 DE-IRGs were downregulated while 190 genes were upregulated in THCA samples (Fig. 2B, E). Subsequently, WGCNA was performed to construct a weighted co-expression network based on the DE-IRGs expression matrix. We used the soft-thresholding power of β = 11 to achieve a scale-free network in the present study. Two gene modules (turquoise and grey modules) were identified. Among two modules, grey module (r = 0.62, p = 1e−62) showed the strongest correlation with THCA tissues (Fig. 3A). Therefore, we regarded the grey module as THCA-specific module and 271 DE-IRGs were identified for subsequent analysis.

### Construction of immune-related eRNAs risk signature

**Fig. 4:**
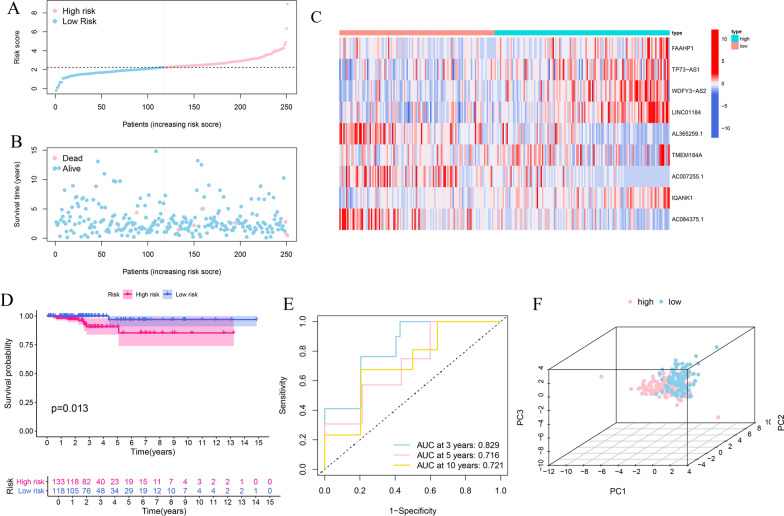
Risk score analysis of the prognostic signature in the training cohort. **A**–**C** The risk score distribution, survival status, expression heatmap of nine immune-related eRNAs between high- and low-risk groups in the training set. **D** Kaplan–Meier survival curve analysis of OS in high- and low-risk groups. **E** Time-dependent ROC curve analysis of the prognostic signature. **F** PCA analysis in high- and low-risk groups

The expression matrixes of 1565 eRNAs among 1584 eRNAs and 271 DE-IRGs in grey module were extracted from the TCGA-THCA samples. A total of 125 immune-related eRNAs were obtained between 1565 eRNAs and 271 DE-IRGs using Pearson correlation analysis with |R| > 0.4, p < 0.001. The Univariate Cox regression analysis was conducted in the training cohort to explore the prognostic value of 125 immune-related eRNAs. Sixteen eRNAs were significantly correlated with OS in the training cohort (p < 0.05) (Fig. 3B). The expression levels of 16 prognostic eRNAs were presented in heatmap (Fig. 3C). The LASSO Cox regression analysis was performed to build the prognostic signature in the training set based on the 16 prognostic genes. According to the minimum criteria, a 9-gene risk signature consisting of FAAHP1, TP73-AS1, WDFY3-AS2, LINC01184, AL365259.1, TMEM184A, AC007255.1, IQANK1 and AC084375.1 was constructed (Fig. 3D, E). The immune-related eRNAs co-expression network was constructed based on the 9 genes (Fig. 3F). The risk score was calculated according to the following formula: risk score = (0.1087* expression level of FAAHP1) + (0.0749* expression level of TP73-AS1) + (0.3578* expression level of WDFY3-AS2) + (0.3184* expression level of LINC01184) + (− 0.4084* expression level of AL365259.1) + (0.1747* expression level of TMEM184A) + (− 0.0476* expression level of AC007255.1) + (0.3280* expression level of IQANK1) + (− 0.1432* expression level of AC084375.1). Risk score of each patient in the training cohort was calculated and then patients were separated into high and low-risk subgroups according to the median risk score (Fig. 4A). Patients with high-risk group had more deaths and a shorter survival time than those in low-risk group (Fig. 4B). The heatmap revealed the expression patterns of 9 eRNAs between two different risk subgroups (Fig. 4C). The PCA analysis indicated that high and low-risk patients were well separated into two clusters (Fig. 4F). Kaplan-Meier survival curve indicated that high-risk group had a significantly poorer OS than low-risk groups (p = 0.013) (Fig. 4D). ROC analysis was conducted to evaluate the predictive accuracy of the risk score and the areas under the ROC curve (AUC) were 0.829 for 3 year, 0.716 for 5 year, and 0.721 for 10 year (Fig. 4E).

**Fig. 5:**
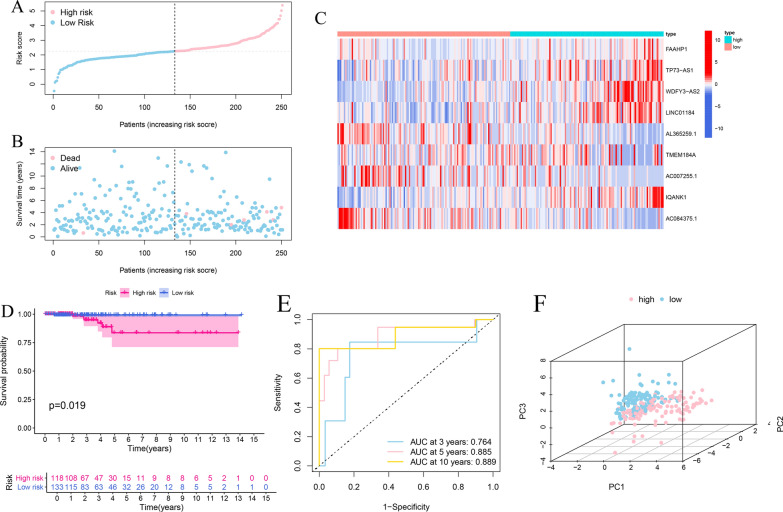
Prognostic assessment of the risk signature in test cohort. **A**–**C** The risk score distribution, survival status, expression heatmap of nine immune-related eRNAs between high- and low-risk groups in test set. **D** Kaplan–Meier survival curve analysis for comparison of OS in high- and low-risk groups. **E** Time-dependent ROC curve analysis of the risk signature. **F** PCA plot of high- and low-risk groups

Furthermore, the predictive capability of the risk signature was validated using test cohort and total cohort. The risk score of each patient was calculated according to the same formula as the training cohort. Patients in test cohort and total cohort were also classified into high- and low-risk groups using the same median score in the training set. The OS of the patients with high-risk score was lower than that of the low-risk groups in the test cohort (p = 0.019) (Fig. 5D). The AUC values of the ROC curve were 0.764 at 3 year, 0.885 at 5 year, and 0.889 at 10 year (Fig. 5E). We ranked the risk scores of patients in the test set and analyzed their distribution, survival status, and the expressions heatmap of nine biomarkers between high- and low-risk groups (Fig. 5A–C). The PCA plot indicated satisfactory separation in different risk subgroups (Fig. 5F).

**Fig. 6:**
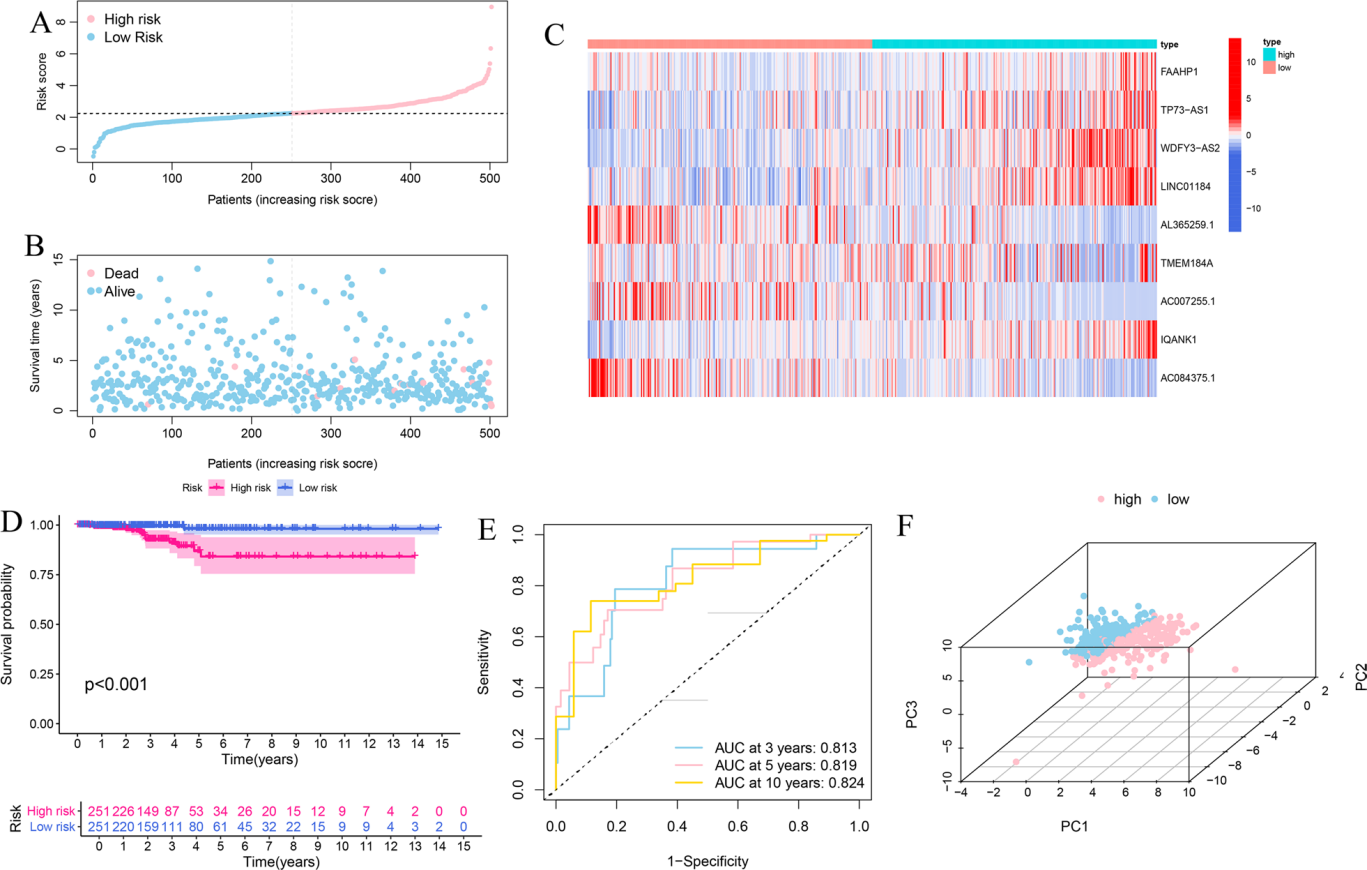
Validation the risk signature in total cohort. **A**–**C** The risk score distribution, survival status, expression heatmap of nine immune-related eRNAs between high- and low-risk groups in total cohort. **D** Kaplan–Meier survival curve analysis for high- and low-risk groups. **E** Time-dependent ROC curve analysis of the risk signature. **F** PCA analysis of high- and low-risk groups in total set

The risk score distribution, survival status and the expression heatmap of nine eRNAs in the total cohort were displayed in Fig. 6A–C. The PCA analysis showed that risk genes could separate two risk groups (Fig. 6F). The result of the OS suggested that high-risk groups had a poorer prognosis compared with low-risk groups (Fig. 6D). The ROC analysis showed that the risk signature exhibited a reliable predictive capability (AUC = 0.813 in 3 year, 0.819 in 5 year and 0.824 in 10 year) (Fig. 6E). These results indicated that the risk signature was a reliable index.

### Clinical value of risk signature

**Fig. 7:**
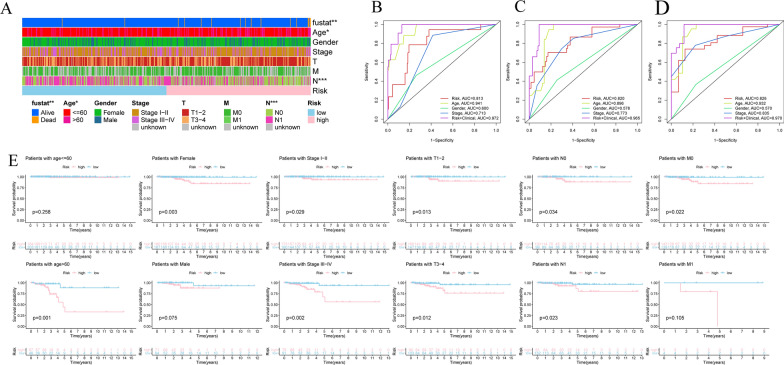
Correlation of the risk score and clinical characteristics in THCA patients. **A** Heatmap of the association between risk score and clinical clinicopathologic features (*p < 0.05, **p < 0.01, ***p < 0.001). (B-D) ROC curves of the risk score and clinical characteristic for 3 year, 5 year, 10 year. **E** Stratified survival analysis of high- and low-risk groups based on age, gender, clinical stage, TNM stage

The possible relationships between risk signature and clinicopathological features were explored and the heatmap showed the distributions of clinical characteristics (survival status, age, gender, clinical stage and TNM stage) in different risk subgroups. The risk score was significantly associated with survival status, age and N stage (p < 0.05) (Fig. 7A). In order to examine the predictive effects of the risk signature, all patients with THCA were separated into different subgroups according to different clinical characteristics, including age, gender, pathological tumor stage and TNM stage. The K-M survival curve suggested that high risk score predicted poorer prognosis in age > 60 (p = 0.002), gender (p = 0.003 in female), stage (p = 0.029 in stage I–II and p = 0.002 in stage III–IV). Similar results were also observed in T1–2 (P = 0.013) and T3–4 groups (P = 0.012), stage N0 (P = 0.034) and stage N1 groups (P = 0.023), and M0 stage (P = 0.022) (Fig. 7E). Furthermore, ROC analysis was conducted to evaluate the predictive performance based on the risk score and clinicopathological characteristics. The AUCs of the risk score (0.813), age (0.941) and stage (0.713) at 3 year, risk score (0.820), age (0.896) and stage (0.773) at 5 year, and risk score (0.826), age (0.932) and stage (0.835) at 10 year, indicating a better predictive capability (Fig. 7B–D). More accurate prediction power was observed after combining risk score with other clinical features.

### Independent prognostic value of the risk signature

**Fig. 8:**
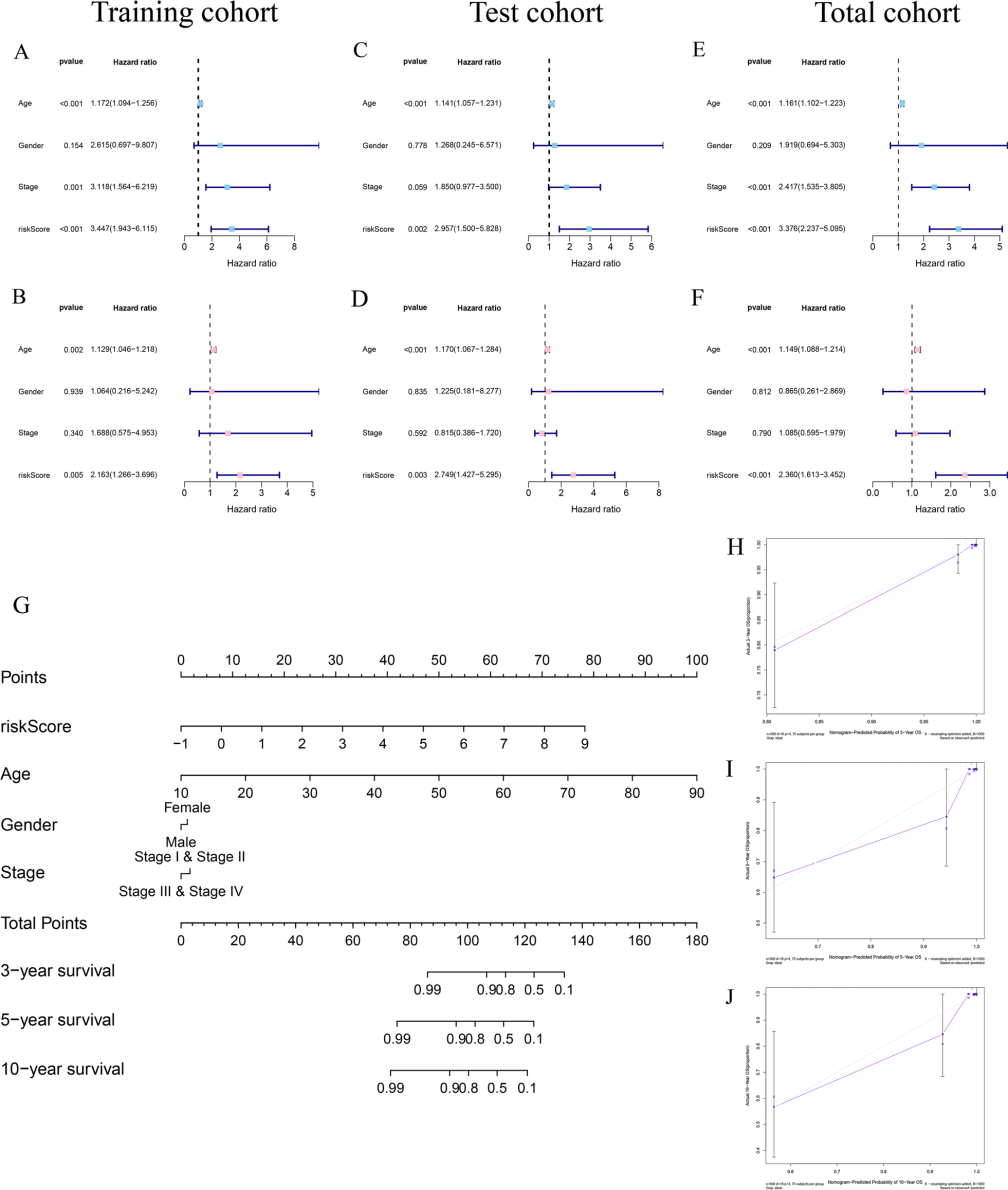
Independent prognosis analysis and clinical value of the risk signature. **A**–**B** The univariate and multivariate Cox regression analysis of the risk score and clinical parameters for OS in the training cohort. **C**–**D** Univariate and multivariate Cox regression analysis for test cohort. **E**–**F** Univariate and multivariate Cox regression analysis of OS in total cohort. **G** Nomogram integrating risk score and clinical features for predicting OS in total set. **H**–**J** The calibration curve of the nomogram for predicting the probabilities of 3 year, 5 year, and 10 year OS

Univariate and multivariate Cox proportional hazards regression analyses were performed in training cohort, test cohort and total cohort to evaluate the independent prognostic value of risk score with clinical factors, including age, gender and pathological stage for THCA patients. Univariate Cox regression analysis showed that age and risk score were significantly associated with OS [training cohort: hazard ratio (HR) = 3.447, 95% confidence interval (CI) = 1.943−6.115, *P* < 0.001; test cohort: HR = 2.957, 95% CI = 1.500−5.828,* P* = 0.002; total cohort: HR = 3.376, 95% CI = 2.237−5.095, *P* < 0.001] (Fig. 8A, C, E). After including other confounding variables in multivariate Cox regression analysis, the risk score was further identified as an independent prognostic factor [training set: HR (95% CI) = 2.163 (1.266−3.696), *P* = 0.005; test set: HR (95% CI) = 2.749 (1.427−5.295), *P* = 0.003; total set: HR (95% CI) = 2.360 (1.613−3.452), *P* < 0.001] (Fig. 8B, D, F).

Subsequently, we developed a prognostic nomogram to provide a quantitative analysis tool for predicting prognosis in patients with THCA based on the risk score and clinicopathological features (age, gender and stage) (Fig. 8G). The calibration curves of 3 year, 5 year and 10 year OS demonstrated an ideal consistency in predictive and actual survivals (Fig. 8H–J).

### Functional enrichment analysis of DEGs between different risk groups

**Fig. 9:**
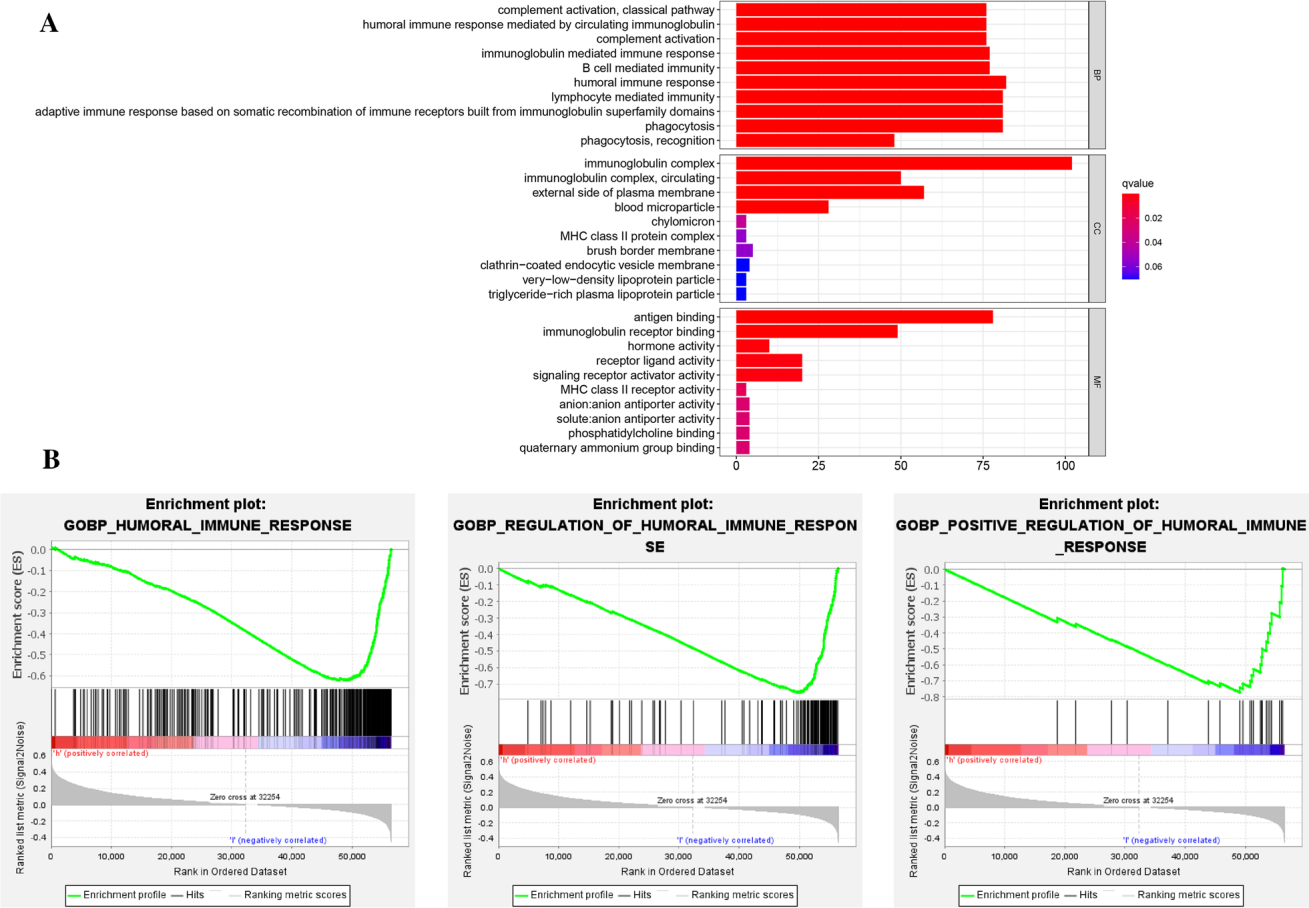
Functional enrichment analysis of DEGs between different risk subgroups. **A** GO enrichment analysis of DEGs between high- and low-risk groups. **B** GSEA using immune gene set revealed that DEGs were mainly enriched in low-risk groups with immune-associated biological processes

To further investigate the differences in gene functions between different risk subgroups, we identified 355 DEGs (201 downregulated genes and 154 upregulated genes in high-risk group) between high- and low-risk groups (Additional file 4: Table S2). The result of GO functional analysis suggested that the DEGs were mainly enriched in humoral immune response, immunoglobulin complex, and antigen binding (Fig. 9A). Additionally, GSEA indicated that humoral immune response, regulation of humoral immune response, and positive regulation of humoral immune response were significantly involved in low-risk group (Fig. 9B).

### Comparison of the tumor immune microenvironment in different risk subgroups

**Fig. 10:**
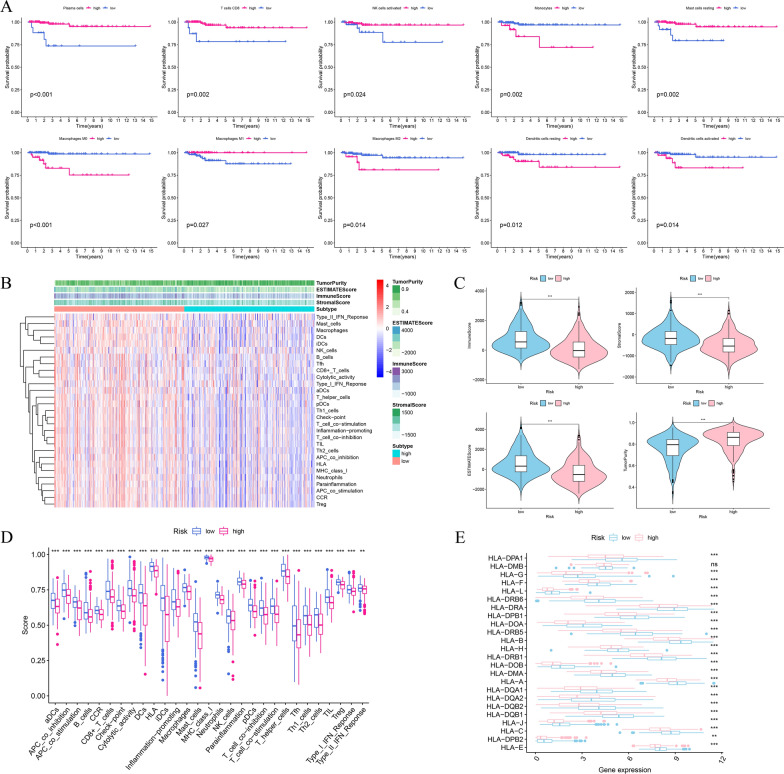
Analysis of tumor immune microenvironment between different risk groups. **A** K-M survival analysis of immune cells infiltration. **B** Heatmap of immune cells infiltration and immune function in TCGA. **C** Comparison of immune score, stromal score, ESTIMATE score, and tumor purity between high- and low-risk groups. **D** The ssGSEA score of 16 kinds of immune cells and 13 immune pathways between high- and low-risk groups. **E** The expression levels of HLA family genes in high- and low-risk groups

To explore the association between risk signature and tumor immune microenvironment (TIME), we calculated the relative proportion of each kind of immune cells among THCA patients based on the RNA-sequencing data using CIBERSORT algorithm. The proportions of 21 kinds of tumor-infiltrating immune cells in the low- and high-risk groups were displayed in Additional file 1: Figure S1A. The bar plot indicated that the proportions of dendritic cells resting and T cells CD4 memory activated were significantly higher in low-risk samples compared to high-risk patients (p < 0.05) (Additional file 1: Figure S1B). The infiltrating ratios of Plasma cells, T cells CD8, NK cells activated, Monocytes, Mast cells resting, Macrophages M0, Macrophages M1, Macrophages M2, Dendritic cells resting, and Dendritic cells activated were significantly associated with OS (p < 0.05) (Fig. 10A). The higher infiltrating abundance of Monocytes, Macrophages M0, Macrophages M2, Dendritic cells resting, and Dendritic cells activated were tend to have a poorer OS, while high levels of Plasma cells, T cells CD8, NK cells activated, Mast cells resting, and Macrophages M1 were correlated with better OS.

We also calculated the immune score, stromal score, ESTIMATE score, and tumor purity in each THCA sample to investigate the differences of the TIME between high- and low-risk groups based on ESTIMATE algorithm. We performed ssGSEA to quantify the enrichment scores of 16 kinds of immune cells and the activity of 13 types of immune-related signaling pathways in THCA samples. The heatmap showed 29 immune-related gene sets for THCA patients and correlation between different risk subgroups and immune score, stromal score, ESTIMATE score, and tumor purity (Fig. 10B). The immune score, stromal score and ESTIMATE score were significantly higher in low-risk groups than in high-risk groups (p < 001), while the tumor purity was lower in low-risk groups (p < 001) (Fig. 10C). Moreover, 29 immune-related gene sets were all significantly upregulated in low-risk groups (p < 0.01) (Fig. 10D). Furthermore, the expression levels of the HLA family genes were higher in the low-risk groups except for HLA-DMB (p < 0.01) (Fig. 10E).

**Fig. 11:**
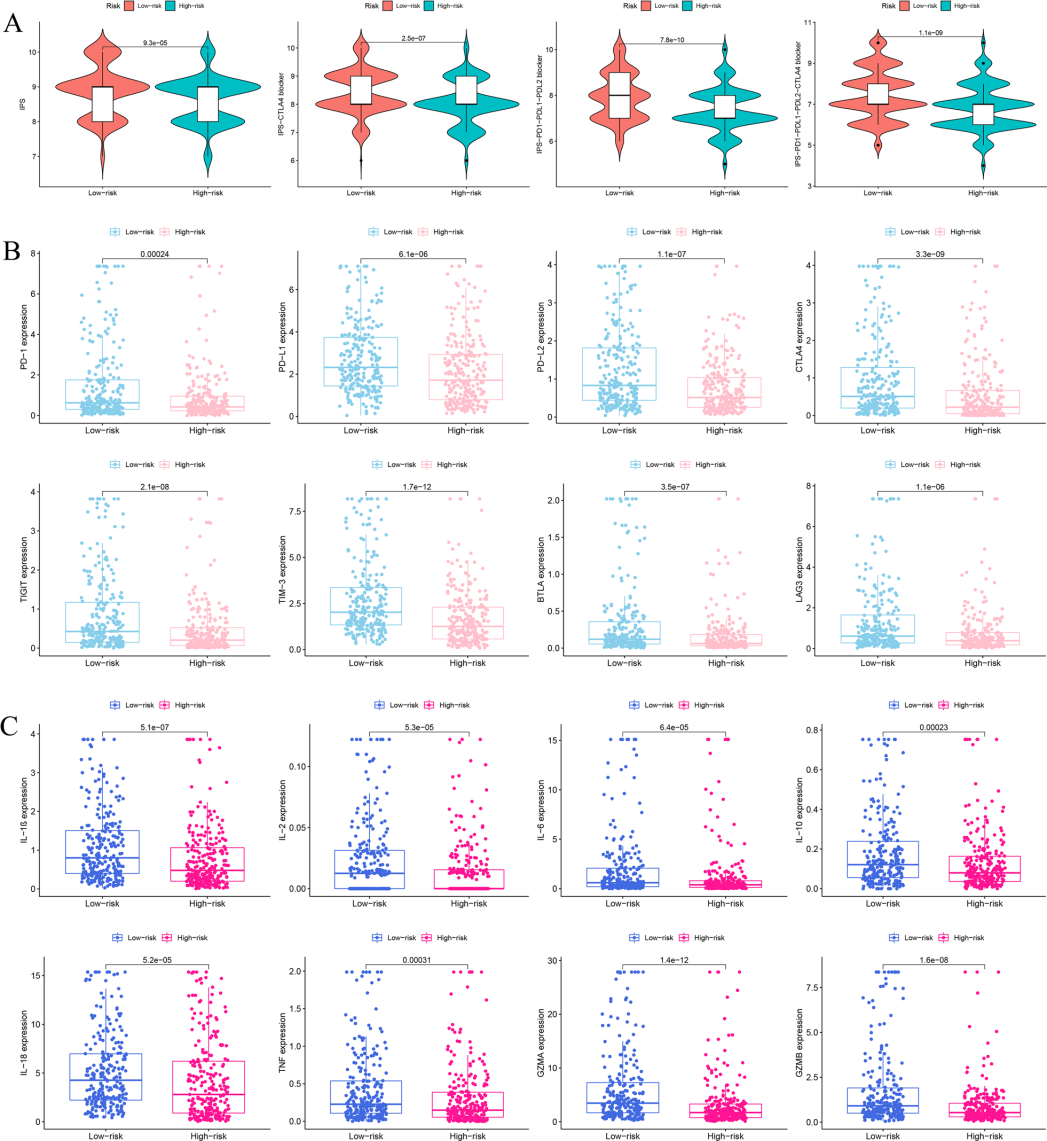
Immune checkpoint expression analysis. **A** The correlation between IPS and risk score in THCA patients. **B** Comparison of the expression levels of immune checkpoint between high- and low-risk groups. **C** The expression levels of cytokines between high- and low-risk groups

The correlation between IPS and prognostic risk signature in THCA was explored to predict the patients’ response to immune checkpoint inhibitors (ICIs). The IPS, IPS-CTLA4, IPS-PD1/PD-L1/PD-L2, and IPS-PD1/PD-L1/PD-L2 + CTLA4 scores were markedly higher in low-risk subgroups (p < 0.001) (Fig. 11A). The expression levels of PD1, PD-L1, PD-L2, CTLA4, TIGIT, TIM-3, BTLA, and LAG3 were significantly higher in low-risk groups (p < 0.001) (Fig. 11B). Furthermore, we also compared the difference in expression levels of cytokines between two different risk subgroups. There were significant differences in the expression levels of interleukin 1 beta (IL-1β), IL-2, IL-6, IL-10, IL-18, tumor necrosis factor (TNF), granzyme A (GZMA), and GZMB in high- and low-risk groups (p < 0.001) (Fig. 11C). These results indicated that low-risk groups patients appeared to have a better opportunity for ICI treatment.

### Validation expression of risk eRNAs in THCA tissues

**Fig. 12:**
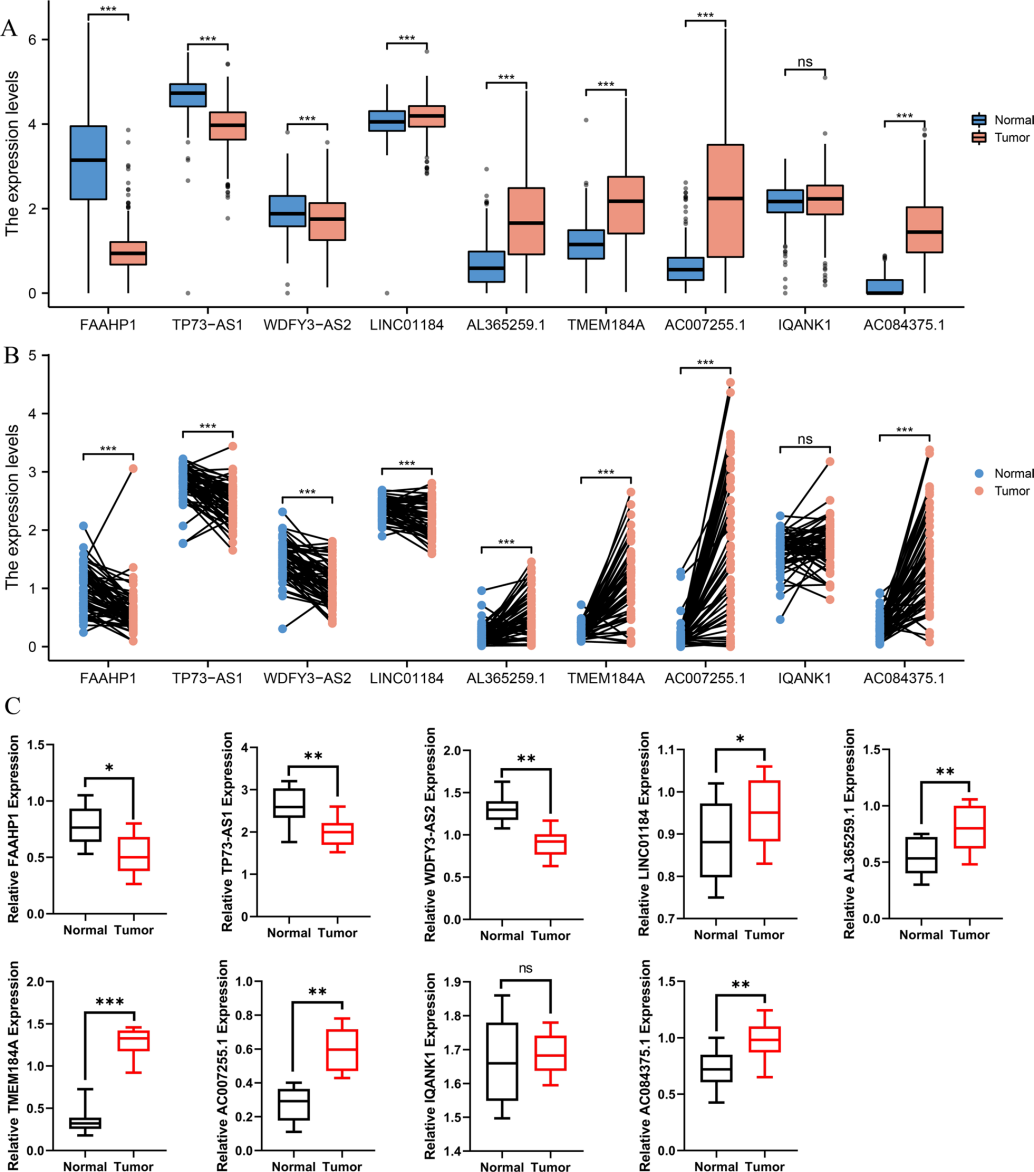
The expression levels of 9 eRNAs in TCGA database and tumor tissues. **A** Expression levels of 9 eRNAs in THCA samples from GTEx database. **B** The levels of these nine eRNAs in paired adjacent normal and tumor samples from TCGA database. **C** The expression levels of these 9 eRNAs in clinical tissues

The expressions of FAAHP1, TP73-AS1, and WDFY3-AS2 were significantly down-expressed, while LINC01184, AL365259.1, TMEM184A, AC007255.1, and AC084375.1 expression were significantly increased in THCA tissues compared with normal samples in TCGA and GTEx databases (p < 0.001) (Fig. 12A–B). Nevertheless, IQANK1 expression showed no statistical difference. The FAAHP1, TP73-AS1, WDFY3-AS2, LINC01184, AL365259.1, TMEM184A, AC007255.1, and AC084375.1 indicated well diagnostic accuracy with the area under the ROC curve (AUC) > 0.7 (Additional file 2: Figure S2). To further validate the expression level of 9 prognostic eRNAs, we analyzed the differential expression levels in normal samples and THCA tissues based on TCGA database aa well as in 62 pairs of thyroid cancer tissues and adjacent normal tissues by qRT-PCR (Fig. 12C). The expression levels were consistent with the results of bioinformatic analysis.

## Discussion

THCA is the most common form of endocrine system tumor, and its incidence has rapidly increased over the past few decades. PTC is the most predominant pathological subtype among all the THCA cases. Most PTC patients usually have a favorable prognosis when surgical treatment, radioactive iodine therapy, and thyroid-stimulating hormone (TSH) suppression treatment are implemented. Nonetheless, more than 10% of PTC patients may suffer local recurrence and distant metastasis after the initial treatment. Therefore, it is essential to explore new potential molecular targets for clinical therapy in order to improve patient outcomes.

TME consists of immune cells and non-immune stromal cells, and immune system was found to make a crucial contribution to cancer development and progression. Cancer immunotherapy has made tremendous progress for some cancer types in recent years.

Predictive or prognostic biomarkers related to the TIME have great promise for identifying novel molecular therapeutic targets and improving cancer patients clinical management of immunotherapy [31].

Long non-coding RNAs (lncRNAs) have emerged as enormous amount and diverse functions in the past decade and have been reported to play important roles in a variety of biological processes, including cell proliferation, death, and tumor growth [32]. Enhancer was described as distal regulatory DNA that regulate the transcription of target genes by interacting with promoters of target genes. Studies suggested that enhancers can transcribe non-coding RNAs, which was been defined as enhancer RNAs (eRNAs) [33]. eRNAs were limited to around 500–2000 bp and had shorter half-lives [34]. Extensive evidences suggested significant roles of eRNAs in tumorigenesis and they involved in various cancer signaling pathways through regulating their target genes. The activation of oncogenic signaling pathways in human cancers often enhanced enhancer activation and production of eRNAs. CELF2 is highly expressed in stomach adenocarcinoma [35]. APH1A is highly expressed in grade-3 hepatocellular carcinoma (HCC). EN1 is highly expressed in breast cancer (BRAC), and ESR1 can increase eRNA transcription in BRAC [36]. TAOK1 is associated with overall survival in clear cell renal cell carcinoma [22, 37]. SCRIB was differentially expressed among lung adenocarcinoma patients [38]. Tumor suppressors-induced eRNAs may implicated in tumor suppression, while oncogene-induced eRNAs can directly promote tumorigenesis. Therefore, eRNAs were closely correlated to malignancy formation and progression.

In our study, we identified 3451 DEGs from 510 THCA samples and 58 normal samples in TCGA cohort, and 362 DE-IRGs (172 downregulated 190 upregulated genes) were extracted. WGCNA was performed to identify THCA-specific immune-related hub genes based on 362 DE-IRGs, and two gene modules (turquoise and grey modules) were obtained. Finally, the grey module was regarded as THCA-specific module. Pearson correlation analysis was used to evaluate immune-related eRNAs. Subsequently, 9-immune-eRNAs prognostic signature (FAAHP1, TP73-AS1, WDFY3-AS2, LINC01184, AL365259.1, TMEM184A, AC007255.1, IQANK1 and AC084375.1) was constructed based on univariate Cox regression analysis and LASSO Cox regression analysis, which was an independent prognostic factor for OS. TP73-AS1, also known as KIAA0495, is abnormally expressed in many cancers [39]. Previous studies indicated that TP73-AS1 could be a key role in regulating HCC cells proliferation and its expression level was associated with poor prognosis of HCC patients [40]. Besides, Wang et al. showed TP73-AS1 also interfered the metastasis and proliferation of ovarian cancer [41]. Of note, we firstly found the expression level of TP73-AS1 is higher in normal thyroid cancer, suggesting that it might have different mechanism in regulating tumor progression comparing other cancers. Increasingly evidences have implicated that lncRNAs participated in the process of cell growth, invasion. Studies showed that overexpressed WDFY3-AS2 suppressed the proliferation, invasion, and epithelial-to-mesenchymal transition (EMT) in ovarian cancer [42]. Furthermore, WDFY3-AS2 also could promote cisplatin resistance by the expression of miR-139-5p/SDC4 in ovarian cancer, which may provide a promising drug target to drug resistance [43]. WDFY3-AS2 participated in the development and progression of oesophageal squamous cell carcinoma (ESCC) by regulating miR-2355-5p/SOCS2 axis, which suggested that WDFY3-AS2 might be an underlying predictor and novel therapeutic target for ESCC patients [44]. Other studies suggested that the potential value of long noncoding RNA WDFY3-AS2 might be novel prognostic biomarker for lung adenocarcinoma (LUAD) [45], glioma [46], and esophageal cancer (EC) [47]. AC007255.1, an immune-related prognostic eRNA, was up-regulated in EC tissues and high expression indicated a poorer prognosis. It was closely related to immune response and infiltration levels of immune cells, such as B cell, dendritic cell and neutrophil [48]. Sui et al. demonstrated that LINC01184 was highly expressed in colorectal cancer, and it could affect the the proliferation and invasion of colorectal cancer cells through the linc01184-miR-331-HER2-p-Akt/ERK1/2 pathway [49]. Subsequently, the risk score was calculated and separated all patients into high- and low-risk subgroups based on median risk score, and Kaplan–Meier survival curves indicated that the high-risk groups had poorer clinical results than that of the low-risk groups. Univariate and multivariate Cox regression analyses showed the risk score was an independent prognostic factor. Additionally, GO functional analysis suggested that the DEGs between different risk groups were mainly enriched in humoral immune response, immunoglobulin complex, and antigen binding. GSEA showed that humoral immune response, regulation of humoral immune response, and positive regulation of humoral immune response were significantly enriched in low-risk group. To further estimate the TIME of the prognostic signature, ESTIMATE and CIBERSORT were performed to estimate the immune score, stromal score, and tumor purity in THCA sample. More dendritic cells resting and T cells CD4 memory activated were significantly infiltrated in TIME of low-risk group. In addition, patients in the low-risk groups had higher immune score, stromal score and ESTIMATE score than in high-risk group. We applied ssGSEA to assess the immune status of the risk signature, the results suggested that 29 immune-related cells were significantly up-regulated in low-risk group. At the same time, the expression levels of cytokines and immunosuppressor molecules (PD1, PD-L1, PD-L2, CTLA4, TIGIT, TIM-3, BTLA, and LAG3) were significantly higher in low-risk groups, implying more tumor immunogenicity in the low-risk group. The good response of ICIs might be one of the reasons for the good clinical outcome in the low-risk group. Therefore, patients with low-risk scores might be more likely to benefit from ICI treatment.

The present study has some limitations. Our data was retrieved from TCGA public database instead of our own cohort, the predictive power of the prognostic signature should be validated using an external validation cohort, such as GEO cohort. Nevertheless, information on overall survival of THCA patients was unfortunately largely lacking in the GEO databases. Moreover, some basic experiments should be performed to further validate our bioinformatics analysis results in the future. The efficacy of immunotherapy in THCA patients was needed to be validated in large clinical trials.

## Conclusion

Taken together, we are the first to construct an immune-related eRNAs prognostic signature to efficiently predict the survival and prognosis with high specificity for THCA patients. Significant differences were founded between prognostic signature and TIME, all patients with different risk levels exhibited different response to immunotherapy. This study could provide a novel insight into a potentially novel prognostic prediction and offer opportunity for individualized immunotherapy of THCA patients in future studies.

## Supplementary Information

**Additional file 1: Figure S1.** Tumor-infiltrating immune cells in different risk subgroups. **A** The abundance of 21 immune cells between high- and low-risk groups. **B** Differences in fractions of tumor-infiltrating immune cells between two risk subgroups.

**Additional file 2: Figure S2.** The diagnostic values of 9 eRNAs for THCA patients.

**Additional file 3: Table S1.** Premier sequences for qRT-PCR analysis.

**Additional file 4: Table S2.** The differentially expressed genes between high and low-risk group.

## Data Availability

Gene expression profiles, clinical information of THCA in this study are available from the public database (TCGA, https://portal.gdc.cancer.gov/) and Genotype-Tissue Expression Project (GTEx) database (https://www.gtexportal.org/). The immune related genes are acquired from the Immunology Database and Analysis Portal database (ImmPort, https://immport.niaid.nih.gov) and InnateDB (https://www.innatedb.com/) databases. The IPS values are downloaded from The Cancer Immunome Atlas (TCIA, https://tcia.at/home).
