# Supplementary figures and images for: Evaluate the immune-related eRNA models and signature score to predict the response to immunotherapy in thyroid carcinoma
Source: Cancer Cell Int. 2022 Oct 10;22:307. doi: 10.1186/s12935-022-02722-8 (PMC9549686; doi:10.1186/s12935-022-02722-8)

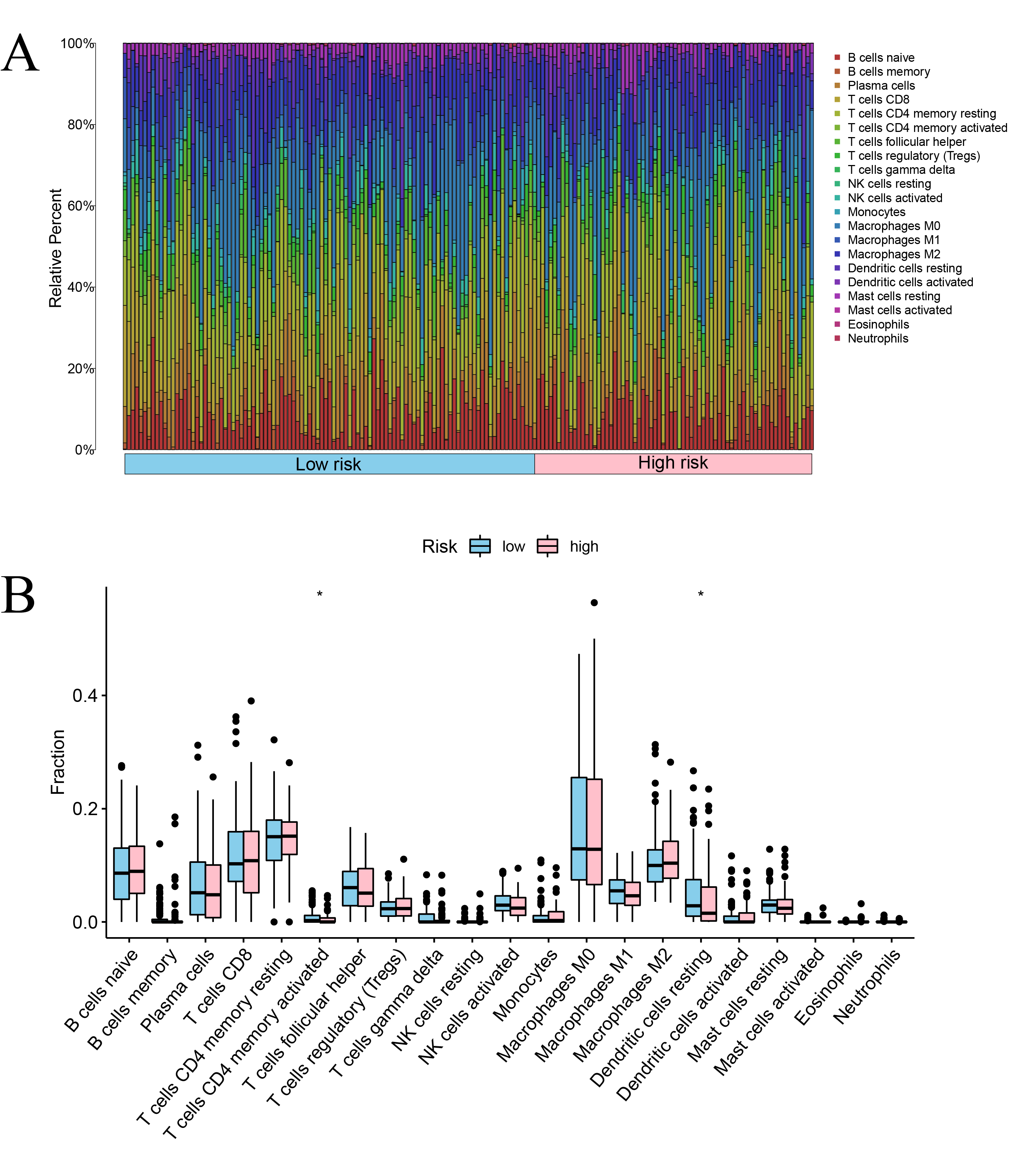

Supplement: Supplementary file 1 — Additional file 1: Figure S1. Tumor-infiltrating immune cells in different risk subgroups. A The abundance of 21 immune cells between high- and low-risk groups. B Differences in fractions of tumor-infiltrating immune cells between two risk subgroups. [file 12935_2022_2722_MOESM1_ESM.tif]

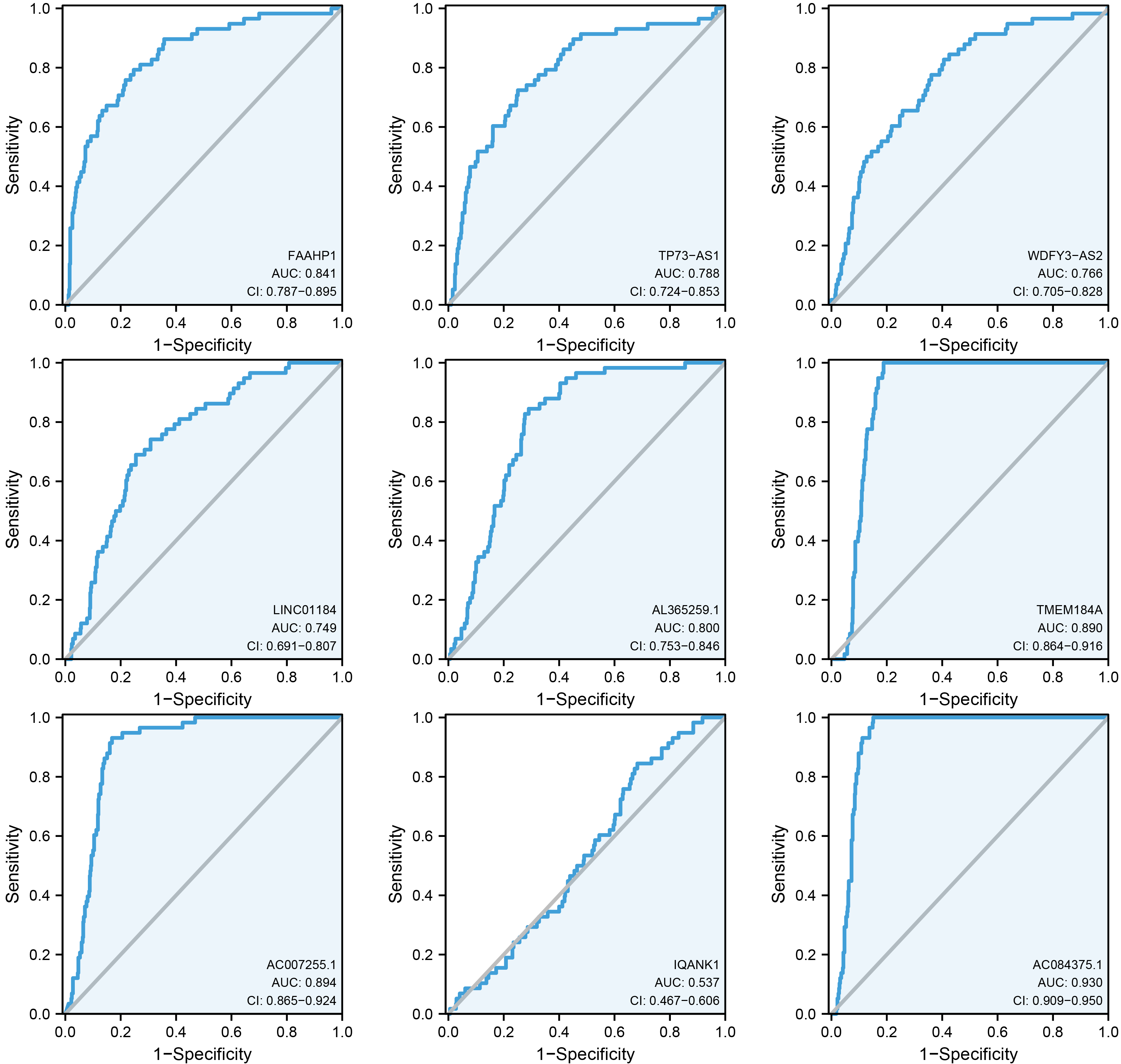

Supplement: Supplementary file 2 — Additional file 2: Figure S2. The diagnostic values of 9 eRNAs for THCA patients. [file 12935_2022_2722_MOESM2_ESM.tif]
